# BluePen Biomarkers LLC: integrated biomarker solutions

**DOI:** 10.4155/fsoa-2016-0031

**Published:** 2016-06-09

**Authors:** Ian A Blair, Clementina Mesaros, Patrick Lilley, Matthew Nunez

**Affiliations:** 1 BluePen Biomarkers LLC, 3401 Grays Ferry Avenue, Building 212, Suite 132, Philadelphia, PA 19146-2701, USA; 2 Penn SRP Center, University of Pennsylvania, Philadelphia, PA 19104-6160, USA; 3 Department of Systems Pharmacology & Translational Therapeutics, University of Pennsylvania, Philadelphia, PA 19104-6160, USA; 4 Emerald Logic, Aliso Viejo, CA 92656, USA; 5 BluePrint Bio, Aliso Viejo, CA 92656, USA

**Keywords:** bioinformatics, biomarkers, lipidomics, metabolomics, proteomics, systems biology

## Abstract

BluePen Biomarkers provides a unique comprehensive multi-omics biomarker discovery and validation platform. We can quantify, integrate and analyze genomics, proteomics, metabolomics and lipidomics biomarkers, alongside clinical data, demographics and other phenotypic data. A unique bio-inspired signal processing analytic approach is used that has the proven ability to identify biomarkers in a wide variety of diseases. The resulting biomarkers can be used for diagnosis, prognosis, mechanistic studies and predicting treatment response, in contexts from core research through clinical trials. BluePen Biomarkers provides an additional groundbreaking research goal: identifying surrogate biomarkers from different modalities. This not only provides new biological insights, but enables least invasive, least-cost tests that meet or exceed the predictive quality of current tests.

**Figure 1.**
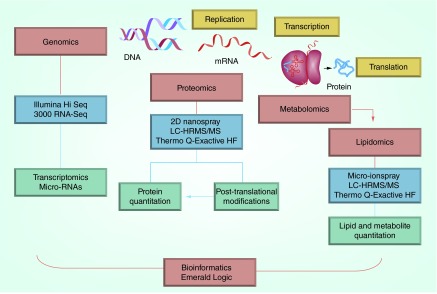
**BluePen Biomarkers systems biology approach to biomarker discovery and validation.**

BluePen Biomarkers was established in April 2016 in Philadelphia PA through a collaboration between the University of Pennsylvania's Center for Innovation, Blueprint Bio and Emerald Logic from Aliso Viejo (www.bluepenbio.com). The company was founded by experts from the field of biomarker discovery and validation at the University of Pennsylvania, which complements the bioinformatics expertise provided by Emerald Logic from Aliso Viejo (CA, USA). A major goal of the company is to address the problem of poorly predictive biomarkers that was identified by George Poste in his provocative 2011 article in *Nature* [1]. A coordinated systems biology approach has been developed to replace the patchwork of different technologies that are currently used to identify biomarkers [1]. Our approach involves the use of integrated multi-omics technologies as described below:

BluePen Biomarker's team of management and scientific advisors comprises leading biomedical scientists who provide a coordinated approach to fully integrated biomarker solutions;
Genomics, proteomics, metabolomics and lipidomics research is conducted in BluePen Biomarkers’ laboratories using state-of-the-art instrumentation;
The products of multi-omics research are integrated using sophisticated bioinformatics based on combinatorial signal processing and analytic approaches developed by Emerald Logic to provide a fully integrated biomarker solution (Figure 1);
The fundamental conviction of the company's founders is that any intervention that has a biologic outcome will have a detectable predictive biomarker signature. The challenge is to detect that signature;
The comprehensive multi-omics systems biology platform available at BluePen Biomarkers coupled with Emerald Logic's innovative signal processing approach provides a powerful solution to the biomarker needs of academia as well as pharmaceutical and biotech industries (Figure 1);
The substantial expertise available within the company provides a unique resource for assessing potential clinical biomarker needs;
Biomarker discovery and validation can be tailored to the particular project under consideration;
BluePen Biomarkers now provides a commercial multi-omics platform that can quickly yield highly predictive biomarkers.

## Discussion

Whole blood is normally provided to BluePen Biomarkers for genomics analysis; whereas serum is normally supplied for proteomics, metabolomics and lipidomics analyses. However, buffy coat or peripheral blood monocytes (PBMCs) are sometimes provided for proteomics analyses and platelets are sometimes provided for metabolism studies or specific mitochondrial protein quantification [2–4]. BluePen Biomarkers major services are summarized below:

Genomics analyses generally involve RNA sequencing (RNA-Seq), which is sometimes known as whole transcriptome shotgun sequencing. RNA-Seq, which is conducted by next-generation sequencing (NGS) using an Illumina HiSeq 3000 instrument, has largely replaced microarray analysis for the quantification of mRNA transcripts in a biofluid sample at a given moment in time. This can provide biomarker signatures of particular disease states. RNA-Seq can also be used to assess alternative gene-spliced transcripts, post-transcriptional modifications, gene fusion, mutations/single-nucleotide polymorphisms, different populations of RNA, total RNA, micro-RNA and transfer-RNA;
Proteomics analyses normally involve targeted quantification of the major proteins that are identified as being upregulated by the RNA-Seq data. Analyses are conducted using nanospray ionization coupled with two-dimensional ultraperformance liquid chromatography/high-resolution mass spectrometry (UPLC-HRMS/MS) coupled with parallel reaction monitoring (PRM). These studies are conducted on a Thermo Q Exactive HF Hybrid Quadrupole-Orbitrap mass spectrometer operating in a targeted mode. Validation of differentially regulated proteins is conducted using rigorous stable isotope dilution UPLC-HRMS. Stable isotope-labeled protein standards are generated using stable isotope labeling by amino acids in cell culture (SILAC) based methodology [5];
Metabolomics and lipidomics analyses are conducted on serum samples that have been extracted using Bligh and Dyer methodology [6,7]. For biomarker discovery, the aqueous phase is subjected to metabolomics analysis and the organic phase is subjected to lipidomics analysis. Both analyses are conducted using heated electrospray ionization (HESI) coupled with UPLC-HRMS/MS on a Thermo Q Exactive HF Hybrid Quadrupole-Orbitrap mass spectrometer. Metabolites and lipids that are differentially expressed between cases and controls are thoroughly characterized by comparison of UPLC-HRMS/MS characteristics with authentic samples either obtained commercially or synthesized in house;
Stable isotope-labeled metabolite and lipid standards are generated using stable isotope labeling by essential nutrients in cell culture (SILEC) methodology [8–10]. There are potentially >100,000 lipids present in serum, although Lipid Maps has only identified some 48,360 lipids (www.lipidmaps.org). This means that BluePen Biomarkers can potentially discover lipid biomarkers where no standards are available to confirm structures. In this case, total synthesis is conducted using the extensive synthetic expertise that is available within the company so that absolute structural confirmation can be conducted before embarking on extensive clinical studies;
Bioinformatics analyses are conducted by Emerald Logic using a proprietary analytic platform that they have developed. This will identify signals in mRNA, protein, metabolite and lipid expression between cases and controls. An optimal panel of biomarkers will then emerge with maximal clinical specificity and sensitivity for a particular indication.

Developing clinically useful biomarkers in biofluids is a complex process that often results in failure [1]. Therefore, it is important that the biomarker discovery studies are conducted in appropriate biofluids and that they are followed by rigorous bioanalytical validation [11]. This involves establishing limits of quantification and estimation of the errors that are introduced by sample handling analogous to methods that are used in the validation of drug assays [12]. The bioanalytical validation is an important first step that is conducted by BluePen Biomarkers before clinical validation is conducted using precious patient samples. As a result, time-consuming and expensive clinical validation studies do not fail as a result of bioanalytical issues and so this rigorous approach ends up being highly cost-effective. Biomarker validation studies require quality control samples to be prepared in the relevant biofluid that is being used. For endogenous biomarkers this can be challenging because it is likely to already be present in the biofluid. In addition, sophisticated methods are required to determine whether an endogenous signal is actually the analyte of interest but not interfering substances with similar physicochemical properties. To best distinguish the target analyte from chemical background peaks, assays have to be conducted with the maximal specificity and sensitivity that is possible using stable isotope analogs as internal standards. Gas chromatography (GC)- and LC-MS are the two most widely used instrument platforms to employ stable isotope dilution methodology. LC-MS is more applicable to the analysis of a wider range of biomarkers than GC-MS and is also inherently easier to use for rigorous validation. Consequently, BluePen Biomarkers uses bioanalytical platforms for proteomics, metabolomics and lipidomics analyses that are all based on LC-MS methodology.

Quantitative biomarker studies generally require the most sensitive means of detection possible [7,11]. The new generation of high-resolution mass spectrometers based on the Thermo Q-Exactive platform can provide data using either the HR selected ion monitoring (SIM) mode or the HRPRM mode. BluePen Biomarkers uses both of these techniques on their Thermo Q-Exactive HF instruments. The specificity of this approach to biomarker analysis arises from the ability to monitor masses with an accuracy of 0.001 Da and the sensitivity derives from the unique capabilities of the Orbitrap that is installed in the HF instrument. A stable (heavy) isotope labeled internal standard is used to establish the presence of an endogenous analyte using both the LC retention time and accurate mass selection of the Q-Exactive HF mass spectrometer. This level of specificity cannot be attained with any other bioanalytical technique employed for biomarker analysis.

An authentic stable isotope-labeled analog of a biomarker is identical to the endogenous molecule, except for its mass [11]. The response ratio between the unlabeled biomarker and its labeled internal standard is then interpolated into a standard curve in order to calculate the absolute amount of a biomarker analyte in the unknown sample. Variations of this technique are also used (most extensively in proteomics) where metabolically labeled proteins can be used at unknown concentrations for relative quantification purposes [13–15]. If necessary, absolute protein quantification can also be performed using appropriate protein standards [16,17]. In all cases, stable isotope internal standards offer the means to verify the presence of the analyte and normalize experimental variables such as sample storage and matrix suppression. Differential ionization cannot occur between an identified biomarker and its stable isotope analog internal standard in the source of the mass spectrometer. This minimizes suppression effects even when chromatographic retention times vary with biofluid samples from different individuals. It is therefore impossible to standardize the amount of suppression occurring within any particular sample. Stable isotope analog internal standards also compensate for the selective binding to active sites on glassware or other surfaces that can occur during extraction and chromatography, preventing significant losses of the biomarker being analyzed. Finally, in some cases, compound enrichment is required to improve the analytical performance, as we showed for amyloid β-peptides [18] and DNA-adducts [19,20], which both required immunoaffinity purification. In these cases, stable isotope analog internal standards were essential.

The search for small molecule biomarkers that are predictive of cardiovascular disease, neurodegenerative diseases and cancers has been particularly intense and frustrating [1]. However, BluePen Biomarkers strongly believes that by linking biomarker discovery and validation with the sophisticated bioinformatics provided by Emerald Logic that it will be possible to rationally approach the development of biomarkers for specific diseases.

## Highlights

BluePen Biomarkers was established in April 2016 to provide a coordinated approach to fully integrated biomarker solutions;
The products of multi-omics research are integrated using sophisticated bioinformatics based on combinatorial signal processing and analytic approaches developed by Emerald Logic to provide a fully integrated biomarker solution;
BluePen Biomarkers believes that by linking biomarker discovery and validation with the sophisticated bioinformatics provided by Emerald Logic that it will be possible to rationally approach the development of biomarkers for specific diseases;
RNA-Seq is used to assess the potential utility of transcript profiling for biomarker analysis and upregulation of relevant proteins is confirmed by targeted proteomics;
BluePen Biomarkers uses methods based on UPLC-HRMS for proteomics, metabolomics and lipidomics methods on their Thermo Q-Exactive HF instruments;
This provides the only commercial multi-omics platform currently available that can quickly yield highly predictive biomarkers;
The fundamental conviction of the company's founders is that any intervention that has a biologic outcome will have a detectable predictive biomarker signature.
