# Erratum

**DOI:** 10.2144/fsoa-2016-0031e1

**Published:** 2019-06-05

The following publication of the Company Profile by IA Blair, C Mesaros, P Lilley & M Nunez, (which appeared in a 2016 issue of *Future Science OA* 2[2], FSO124 [2016]; DOI 10.4155/fsoa-2016-0031), has had the title updated to:

Company profile: BluePen Biomarkers LLC – integrated biomarker solutions

The editors of *Future Science OA* would like to sincerely apologize for any confusion or inconvenience this may have caused our readers.

